# Visual prognosis and surgical timing of Ahmed glaucoma valve implantation for neovascular glaucoma secondary to diabetic vitrectomy

**DOI:** 10.1186/s12886-023-02846-z

**Published:** 2023-03-17

**Authors:** Jong Suk Lee, Young Bok Lee, Tae-Woo Kim, Kyu Hyung Park

**Affiliations:** 1 Department of Ophthalmology, Seoul National University Bundang Hospital, Seongnam, Gyeonggi-do 13620 South Korea; 2 Department of Ophthalmology, Seoul National University College of Medicine, Seoul, South Korea; 3 Department of Ophthalmology, Seoul National University Hospital, Seoul National University College of Medicine, Seoul, South Korea

**Keywords:** Diabetic Retinopathy, Glaucoma, Vitrectomy, Intraocular pressure, Visual acuity

## Abstract

**Background:**

Evaluate the visual outcomes of Ahmed glaucoma valve implantation (AGVI) in patients with neovascular glaucoma (NVG) who underwent diabetic vitrectomy and suggest appropriate AGVI timing.

**Methods:**

Medical records of patients who underwent AGVI due to NVG after diabetic vitrectomy were reviewed. Successful intraocular pressure (IOP) control was defined as an IOP between 6 and 21 mmHg. Visual outcome was compared before NVG diagnosis and after AGVI, and the “favorable” visual outcome was defined as a postoperative deterioration in BCVA of less than 0.3 logMAR units compared to those before the development of NVG. Various factors including surgical timing were evaluated to identify the risk factors associated with unfavorable visual outcome.

**Results:**

A total of 35 eyes were enrolled and divided into group 1(medically uncontrolled NVG group, IOP more than 30mmHg, 16 eyes) and group 2(NVG group responded well to the initial non-surgical treatment but eventually required AGVI, 19 eyes). Despite the favorable rate of normalization of post-AGVI IOP (85.7%), 43.8% in Group 1 and 26.3% in Group 2 showed unfavorable visual outcomes. In group 1, delayed surgical timing more than 1 week from the NVG diagnosis showed a significant association with unfavorable visual outcomes *(P* = 0.041). In group 2, poor patient compliance (follow up loss, refuse surgery) was the main factor of unfavorable visual outcomes.

**Conclusion:**

When NVG occurs in patients with proliferative diabetic retinopathy after vitrectomy, physicians should be cautious not to delay the surgical intervention, especially in patients with IOP of 30 or more despite non-surgical treatment. Early AGVI within six days might be necessary to preserve useful vision in these patients.

## Background

Neovascular glaucoma (NVG) is a severe type of secondary glaucoma characterized by the proliferation of fibrovascular membranes in the anterior segment of the eye, usually with poor treatment response and poor visual outcomes [1, 2]. With the increasing morbidities of ischemic vascular disease, including diabetes, the prevalence of NVG has also increased and now accounts for over 30% of refractory glaucoma [3, 4]. The management of NVG includes both the reduction of ischemic drive with pan-retinal photocoagulation (PRP) or intravitreal anti-vascular endothelial growth factor (VEGF) administration and a decrease in intraocular pressure (IOP) with medical therapy and/or surgery.

If IOP-lowering medical therapy is insufficient, proper IOP management can be achieved with surgical options, including cyclodestructive procedures, filtering surgery, and glaucoma drainage device implantation [1, 5, 6]. When physicians can expect the possibility of visual preservation, the Ahmed valve, a flow-restrictive type glaucoma drainage device, has been used as the first choice of surgical intervention in NVG [1, 3, 7, 8].

However, few studies have been conducted regarding the surgical success rate of Ahmed glaucoma valve implantation (AGVI) in NVG. In previous studies, IOP after AGVI was the main criterion for surgical success [3, 8–12]. Because the underlying conditions in patients with NVG are heterogeneous and vision is already poor in many cases before NVG occurs, other factors such as visual acuity and glaucomatous optic nerve damage were not sufficiently considered. In addition, there is no consistent standard for defining the surgical success or failure of NVG, and above all, it is difficult to collect an appropriate cohort [1, 10]. As a result, there is no criterion for the golden time for favorable visual prognosis until AGVI is decided.

Proliferative diabetic retinopathy (PDR), along with ischemic central retinal vein occlusion (CRVO) and ocular ischemic syndrome (OIS), is one of the most common causative conditions, accounting for 33% of NVG [2, 13]. In particular, after pars plana vitrectomy (PPV) for PDR, NVG tends to be more likely to occur [14–17]. In these cases, the subsequent NVG is devastating because PPV was performed to resolve the vision-threatening conditions in the PDR. Moreover, despite the normalization of IOP after AGVI, some cases have unexplained, irreversible central vision loss. It is clinically important to preserve the central vision because PDR patients after successful diabetic vitrectomy usually have better central vision before NVG than severe ischemic CRVO or OIS. This cohort is also appropriate for evaluating the visual prognosis and associated factors of AGVI for NVG.

In this study, we analyzed the effect of AGVI in NVG that occurred after receiving PPV due to PDR. This study is focused on evaluating the effects of the degree and duration of IIOP on visual prognosis in these patients. The therapeutic effect of AGVI was evaluated mainly by focusing on visual outcomes, and the surgical success rate based on IOP was also presented. Other factors that may affect visual prognosis were also evaluated. This article suggests the appropriate surgical timing for AGVI to preserve useful central vision and provides a hypothesis on the possible mechanism of central visual deterioration.

## Methods

### Subjects

**Fig. 1:**
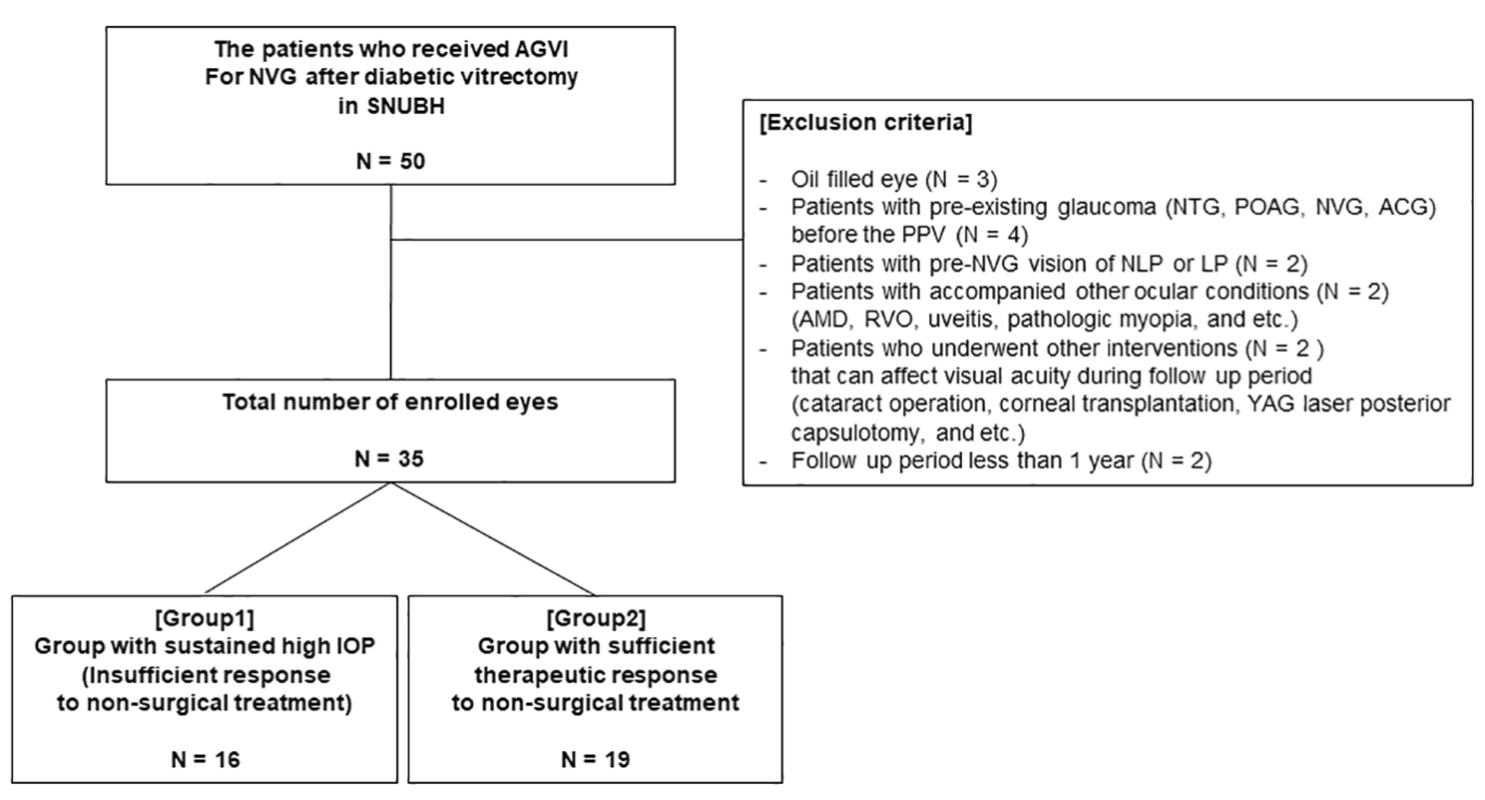
Flow chart for eligible subjects with neovascular glaucoma. Abbreviations: SNUBH, Seoul National University Bundang hospital; AGVI, Ahmed glaucoma valve implantation; NVG, neovascular glaucoma; PPV, pars plana vitrectomy; NTG, normal-tension glaucoma; POAG, primary open-angle glaucoma; ACG, angle-closure glaucoma; NLP, no light perception; LP, light perception; AMD, age-related macular degeneration; RVO, retinal vein occlusion; YAG, Yttrium-Aluminum-Garnet

The medical records of patients who underwent AGVI for NVG after PPV for PDR at Seoul National University Bundang Hospital (SNUBH) between January 2005 and January 2019 were reviewed. NVG was diagnosed by neovascularization of the iris (NVI) and/or iridocorneal angle (NVA), with an IOP elevation of 22 mmHg or more by Goldmann applanation tonometry. Among them, patients with a follow-up period of less than one postoperative (AGVI) year and had been diagnosed with any type of glaucoma (normal-tension glaucoma, primary open-angle glaucoma, preexisting NVG, angle-closure glaucoma including post-PPV angle-closure event) were excluded. Furthermore, patients with other ocular diseases (age-related macular degeneration, retinal vein occlusion, uveitis, and pathologic myopia) or a history of interventions (cataract operation, corneal transplantation, and YAG laser posterior capsulotomy) that can affect visual acuity during the post-AGVI follow-up period were excluded from the analysis. Patients with visual acuity less than hand motion before the diagnosis of NVG were also excluded to prevent a floor effect (Fig. 1). This study was approved by the institutional review board (IRB) of SNUBH, Korea (IRB No. B2003/601 − 104) and conducted in accordance with the tenets of the Declaration of Helsinki. The IRB of SNUBH allowed the waiver of informed consent for individual patients due to the retrospective nature of the study and the analysis used anonymous clinical data.

The degree and duration of increased IOP are considered major factors that can affect the final visual prognosis. To evaluate the effect of pre-AGVI IOP on the visual prognosis, the group with consistently high IOP despite non-surgical treatment (MTMT, anti-VEGF injection, and additional PRP) and the group with a good response to initial non-surgical treatment should be classified separately. We classified the patients with sustained high IOP who did not obtain a pre-AGVI IOP less than 30 mmHg after initial non-surgical treatment into group (1) Patients who obtained an IOP less than 30 mmHg at two or more consecutive outpatient visits after initial non-surgical treatment were classified into group (2) In both group, AGVI was administered at the decision of glaucoma specialists.

### Data collection

The following preoperative baseline data, including demographic features, were collected: age, sex, best-corrected visual acuity (BCVA), IOP, preoperative anterior chamber paracentesis, intravitreal anti-VEGF injection, and the number of IOP-lowering medications during the first week after the diagnosis of NVG. Preoperative BCVA was measured in a sufficiently stable state after the recovery from diabetic vitrectomy. Preoperative IOP includes the IOP measured at the time of the NVG diagnosis and the last IOP within the first week after the NVG diagnosis. During the first week, the change in IOP was also calculated to evaluate the treatment response to initial medical treatment.

The following postoperative data were collected: BCVA, IOP, and surgical complications related to AGVI. Postoperative BCVA was selected as the best result of the BCVA measurements during the one year follow-up period after AGVI. The main outcome measures were preoperative and postoperative BCVA and IOP. Comparing preoperative BCVA and postoperative BCVA, a favorable visual outcome was defined as a BCVA that deteriorated to less than 0.3 logMAR unit after AGVI. Surgical success based on IOP was defined as an IOP between 6 and 21 mmHg with or without anti-glaucoma medication. Surgical failure was defined as an IOP > 21 mmHg on maximally tolerated medication at two consecutive visits, additional glaucoma surgery for IOP control, and other devastating complications such as endophthalmitis and hemophthalmos requiring additional operations. For statistical analysis, we used a LogMAR value of 2.3, 2.6, and 2.9 to represent hand motion, light perception, and no light perception, respectively [18]. If both eyes of a single patient were eligible, both eyes were included.

### Statistical analysis

All data were presented as numbers with percentages for categorical variables or the mean ± standard deviation for continuous variables. A comparative analysis was performed between groups 1 and 2 and between the group with favorable visual outcomes and unfavorable visual outcomes in group 1. Comparisons between the groups were performed using the chi-square test, Fisher’s exact test, independent t-test, and Mann Whitney test. The statistical analysis was performed using SPSS version 25.0 (SPSS Inc., Chicago, IL, USA), and *P* values of < 0.05 were considered significant.

## Results

Thirty-five eyes (of 32 patients) were included in this study. The time interval between diabetic vitrectomy and NVG diagnosis was 259.0 ± 313.7 days (Group 1, 303.3 ± 393.5 days and Group 2, 221.7 ± 231.7). Sixteen eyes with sustained high IOP despite initial non-surgical treatment served as group (1) The remaining 19 eyes served as group (2) In group 2, AGVI was performed after an average of six months (176.2 ± 173.2 days) after the diagnosis of NVG, and the reasons for performing the AGVI varied. In this group, AGVI was administered at the decision of glaucoma specialists when glaucomatous optic neuropathy (GON) progressed despite IOP in the early 20s, or when IOP rose again despite sustained non-surgical treatment. In addition to the above criteria, the patient’s preferences, contralateral blindness, and individual social and environmental factors were also considered.

The demographic features and preoperative information of groups 1 and 2 are summarized in Table 1. There were no significant differences in age or sex. Among the initial non-surgical treatments for NVG, the number of IOP-lowering medications was significantly lower in group 1. This difference was due to the patients who received immediate AGVI at the time of NVG. They usually used less than three IOP-lowering eye drops before AGVI. In three patients, emergency AGVI was performed on the same day that NVG was diagnosed because the involved eye was the last eye, or the patient strongly wanted immediate AGVI. Although there were no significant differences in IOPs at the time of NVG diagnosis between the two groups, the last IOP ‘within the first week’ after the diagnosis of NVG was significantly lower in group 2. Intravitreal anti-VEGF injection showed no statistically significant effect on the acute phase IOP reduction.

**Table 1:** Preoperative data from groups 1 and 2 during the first week after neovascular glaucoma diagnosis

		Total (n = 35)		Group 1 (n = 16)		Group 2 (n = 19)		P-value
**Baseline characteristics**								
Age (years)		58.4 ± 10.3		58.4 ± 10.3		51.9 ± 10.2		0.246†
Sex (M/F)		22/13		8/8		14/5		0.179‡
Laterality (R/L)		18/17		7/9		11/8		0.505‡
VA (LogMAR)								
BCVA before NVG diagnosis		0.85 ± 0.62		0.88 ± 0.50		0.82 ± 0.71		0.483†
VA at the time of NVG diagnosis		1.67 ± 0.79		2.13 ± 0.50		1.28 ± 0.71		0.008†
IOP at the time of NVG diagnosis (mmHg)		40.2 ± 8.3		41.3 ± 8.3		39.3 ± 8.2		0.905†
Lens status (Phakic/Pseudophakic)		5/30		3/13		2/17		0.642‡
**Non-surgical treatment and therapeutic response during the first week after NVG diagnosis**								
No. of IOP-lowering medications		2.51 ± 0.69		2.13 ± 0.78		2.84 ± 0.36		0.005†
AC paracentesis (%)		14 (40.0)		6 (37.5)		8 (42.1)		1.000‡
Anti-VEGF injections (%)		27 (77.1)		12 (75.0)		15 (78.9)		1.000‡
Add PRP		5 (14.3)		1 (6.3)		4 (21.1)		0.347‡
Last IOP within the first week (mmHg)		30.2 ± 11.3 *		38.7 ± 8.3 **		22.3 ± 7.2 ***		0.000†
Delta IOP (mmHg) (IOP at the day of NVG diagnosis - IOP at the last day within the first week)		11.3 ± 10.8 *		3.0 ± 7.7 **		19.0 ± 6.8 ***		0.000†
Delta IOP > 10 mmHg (%)		16 (45.7)		3 (23.1)		13 (68.4)		0.000‡

* Except for eight cases who received immediate AGVI or did not visit within one week from NVG diagnosis

** Except for three cases who received immediate AGVI

*** Except for five cases who did not visit within one week from NVG diagnosis

† Calculated using an independent t-test and Mann Whitney test

‡ Calculated using a Chi-Square test and Fisher’s Exact test

Abbreviations: NVG, neovascular glaucoma; M, male; F, female; R, right; L, left; No., number; IOP, intraocular pressure; AC, anterior chamber; AGVI, Ahmed glaucoma valve implantation; Add PRP, additional pan-retinal photocoagulation

Group 1, Group of NVG patients who maintained an IOP of 30 mmHg or more before AGVI despite initial non-surgical treatment including maximum tolerable medical treatment; Group 2, Group of NVG patients who responded well to initial non-surgical treatment but eventually required AGVI.

Postoperative information are summarized in Table 2. The surgical success rate based on the IOP one year after AGVI was 81.3% and 89.5% in groups 1 and 2, respectively. However, although the overall surgical success rate of AGVI based on IOP was 85.7%, the final visual outcomes were unfavorable in 30% of all patients (43.8% and 26.3% in groups 1 and 2, respectively).

**Table 2:** Postoperative data from groups 1 and 2 during the first year from Ahmed glaucoma valve implantation

	Total (n = 35)	Group 1 (n = 16)	Group 2 (n = 19)	P-value
**Post-AGVI BCVA**	1.22 ± 0.79	1.21 ± 0.69	1.25 ± 0.86	0.961†
**Unfavorable visual outcome (%)**	12 (34.3)	7 (43.8)	5 (26.3)	0.311‡
**IOP at the time of AGVI (mmHg)**	36.8 ± 9.36	41.3 ± 7.4	33.1 ± 9.2	0.003†
**Postoperative IOP**				
3 months after AGVI	17.9 ± 7.1	17.8 ± 9.7	18.0 ± 3.6*	0.108†
6 months after AGVI	17.6 ± 6.1**	16.3 ± 3.7*	18.7 ± 7.3*	0.513†
12 months after AGVI	15.2 ± 4.0**	14.5 ± 3.7*	15.7 ± 4.2*	0.327†
**Surgical success at one year based on IOP (%)**	30 (85.7)	13 (81.3)	17 (89.5)	0.642‡
**Complications (%)**				
Anterior chamber hyphema	6 (17.1)	3 (18.8)	3 (15.8)	1.000‡
Tube obstruction	1 (2.9)	1 (6.3)	0 (0)	0.457‡
Vitreous hemorrhage	8 (22.9)	4 (25.0)	4 (21.1)	1.000‡
Early hypotony with choroidal detachment or collapsed anterior chamber	1 (2.9)	1 (6.3)	0 (0)	0.441‡
Endophthalmitis	0 (0)	0 (0)	0 (0)	1.000‡

*Except for one case who underwent re-AGVI

**Except for two cases who underwent re-AGVI

† Calculated using an independent t-test and Mann Whitney test

‡ Calculated using a Chi-Square test and Fisher’s Exact test

Abbreviations: AGVI, Ahmed glaucoma valve implantation; BCVA, best-corrected visual acuity; IOP, intraocular pressure

Group 1, Group of NVG patients who maintained an IOP of 30 mmHg or more before AGVI despite initial non-surgical treatment including maximum tolerable medical treatment; Group 2, Group of NVG patients who responded well to initial non-surgical treatment but eventually required AGVI.

**Fig. 2:**
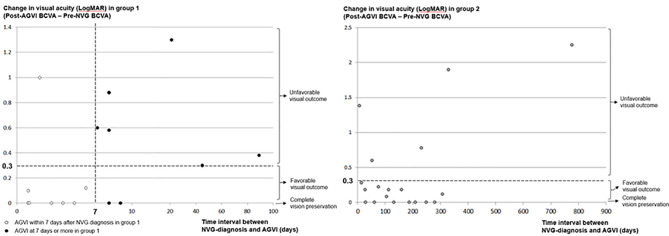
Scatter plot showing the relationship between the timing of AGVI (Ahmed glaucoma valve implantation) and visual outcomes. Groups 1 and 2 were categorized according to the treatment response of initial non-surgical treatment for increased IOP. In group 1, the patients with an unfavorable visual outcome are concentrated in the intervals between NVG diagnosis and AGVI for a week or more (dotted box). In group 2, no clear association was observed between vision prognosis and timing of AGVI

Figure 2 shows the overall visual prognosis according to the time interval between NVG diagnosis and AGVI in group 1 and 2. In group 1, it reveals that the patients with unfavorable visual outcomes are concentrated in the intervals between NVG diagnosis and AGVI for a week or more. On the other hand, no remarkable tendency was observed between visual outcome and AGVI timing in group 2.

In group 1, delayed surgical timing of AGVI for more than one week showed a statistically significant association with unfavorable visual outcomes (*P* = 0.041, Table 3). There were no significant differences in pre-AGVI IOP, baseline BCVA before the diagnosis of NVG, and initial non-surgical treatment options including anti-VEGF injection between the favorable and unfavorable visual outcome groups.

In group 2, the final visual outcome was unfavorable in five patients, but there was no tendency related to AGVI timing. Two of these patients had long-term or multiple follow-up loss events after NVG diagnosis and before AGVI. The other two patients recommended AGVI for an IOP of 40 or more, but the patient’s rejection delayed the AGVI. In the other patient, the IOP decreased to 27 mmHg within one week after the non-surgical treatment, but since the visual acuity had already decreased significantly, AGVI was performed. It is estimated that the period from the outpatient visit to the hospital was delayed after the IOP event occurred. Of the 14 patients with a favorable visual outcome, five patients within one week and 11 patients within three weeks obtained normal IOP with non-surgical treatment. The remaining three patients underwent AGVI due to maintaining IOP in the mid-20s. These 14 patients eventually.

underwent AGVI due to the progression of GON or delayed increase in IOP, but central vision was preserved.

**Table 3:** Factors affecting central vision loss after Ahmed glaucoma valve implantation in Group 1

	Favorable visual outcome* (n = 9)	Unfavorable visual outcome (n = 7)	P-value
**Age (years)**	58.7 ± 11.4	58.0 ± 8.7	0.916†
**Sex (M/F)**	5/4	3/4	1.000‡
**IOP (mmHg)**			
At the time of NVG diagnosis	41.0 ± 8.6	41.6 ± 7.8	0.874†
At the time of AGVI	41.4 ± 7.4	41.0 ± 7.5	0.791†
**BCVA (LogMAR)**			
Before the diagnosis of NVG	0.74 ± 0.42	1.05 ± 0.55	0.238†
At the time of NVG diagnosis	2.23 ± 0.19	2.00 ± 0.23	0.032†
**Early AGVI timing (%)** **(less than one week from NVG diagnosis)**	7 (77.8)	1 (14.3)	0.041‡
**No. of IOP-lowering medications**	2.0 ± 0.94	2.3 ± 0.45	0.678†
**AC paracentesis (%)**	6 (66.7)	6 (42.9)	1.000‡
**Anti-VEGF injections (%)**	5 (62.5)	5 (86.7)	0.585‡
**Add PRP (%)**	0 (0)	1 (14.3)	0.438‡

*Favorable visual outcome : Comparing preoperative BCVA(sufficiently stable BCVA after the recovery from diabetic vitrectomy) and postoperative BCVA (the best result of the BCVA measurements during the one year follow-up period after AGVI), a favorable visual outcome was defined as a BCVA that deteriorated to less than 0.3 logMAR unit after AGVI.

† Calculated using an independent t-test and Mann Whitney test

‡ Calculated using a Chi-Square test and Fisher’s Exact test

Abbreviations: M, male; F, female; IOP, intraocular pressure; BCVA, best-corrected visual acuity; AGVI, Ahmed glaucoma valve implantation; NVG, neovascular glaucoma; AC, anterior chamber; Add PRP, additional pan-retinal photocoagulation

Group 1, Group of NVG patients who maintained an IOP of 30 mmHg or more before AGVI despite initial non-surgical treatment, including maximum tolerable medical treatment.

## Discussion

The prevalence of NVG after diabetic vitrectomy was reported in 4.6-23.6% of cases [14–16, 19]. Concerning changes in intraocular anti-VEGF levels after diabetic vitrectomy, it has been suggested that a high VEGF level could be maintained in the vitreous cavity after vitrectomy for PDR [20–22]. Moreover, vitrectomy with or without a cataract operation can disrupt the structures considered barriers to the diffusion of angiogenic factors to the anterior segment from the posterior segment [23]. Although the effect of vitrectomy (removing the vitreous) or phacovitrectomy itself on the intraocular vasoproliferative substances (VEGF and inflammatory cytokines) and development of NVG has not been established, it is clear that NVG developed after diabetic vitrectomy is clinically important [24–28].

Few studies have been conducted on the prognosis of surgical treatment (AGVI) for NVG after diabetic vitrectomy [17, 29]. They suggested that AGVI is a safe and effective procedure that enables successful IOP control in patients with NVG associated with diabetic vitrectomy. Other studies related to the treatment of NVG also positively evaluated the therapeutic effect of AGVI [3, 8–12]. However, previous studies included cases with heterogeneous underlying ocular conditions and only focused on the normalization of IOP in evaluating the surgical success of AGVI. Detailed visual outcome has not been sufficiently considered due to generally poor visual outcomes and only the proportion of vision less after AGVI was calculated. Because NVG patients after diabetic vitrectomy tend to have better baseline visual acuity before the diagnosis of NVG compared to other conditions, surgical success rate based on visual acuity could be evaluated. Especially, this study focused on patients with unfavorable visual outcomes, even though IOP was well controlled after AGVI.

In addition to normalization of IOP, this study compared the baseline BCVA “before” NVG diagnosis and after AGVI, and the factors affecting visual prognosis were also considered. The overall surgical success rate based on IOP was comparable to a previous study that reported percentages of 83.8% at one year in the NVG after diabetic vitrectomy group [17]. 43.8% and 26.3% had unfavorable visual outcomes in groups 1 and 2, respectively. It is estimated that consistently high IOP may be associated with poor visual prognosis. Especially in group 1, early surgical timing within a week was significantly related to vision preservation (favorable visual outcome). This suggests that close observation should be performed during the first week after NVG diagnosis, and AGVI should be considered without delay in cases with insufficient response to initial non-surgical treatment. Based on our study’s results, we suggest that the IOP change during the first week after NVG diagnosis could be used as a criterion for distinguishing group 1 from group 2. In particular, the change in IOP over the first week can be used as a useful indicator when the standard was set to 10 mmHg (Table 1).

**Fig. 3:**
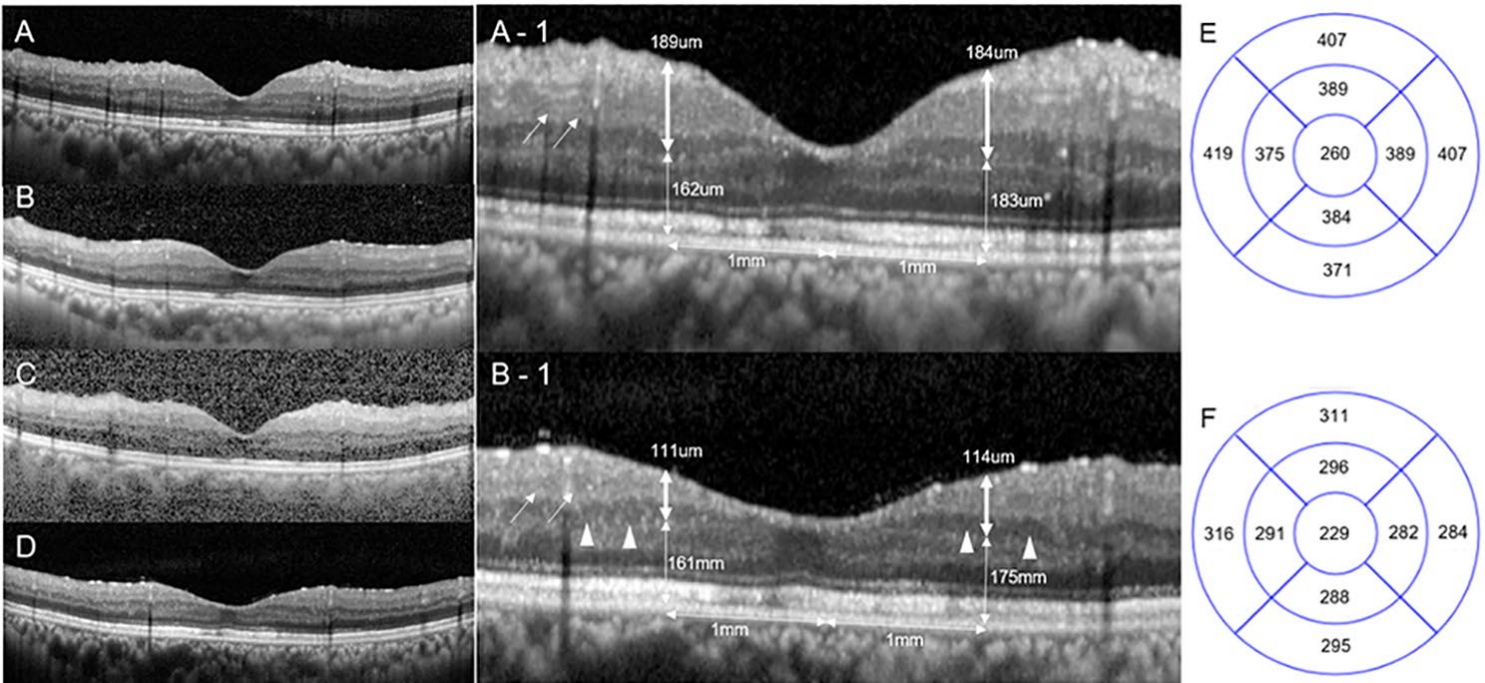
Optical coherence tomographic findings (vertical sections) of a representative case showing ischemic retinal damage due to sustained high IOP during the NVG attack. This patient had undergone AGVI seven days after NVG diagnosis, and their final visual outcome was unfavorable. A is an OCT finding taken one year after diabetic PPV (three months before NVG diagnosis). B and C are OCT findings at the time of NVG diagnosis and three days after AGVI, respectively. Figures B and C shows inner retinal edema and hyper-reflectivity, suggesting retinal ischemia. D is an OCT photograph one year after the occurrence of NVG, and prominent thinning of inner retinal layers is confirmed. A-1 and B-1 are magnified images of A and B, respectively. The inner retinal layer includes the nerve fiber layer, ganglion cell layer, inner plexiform layer, and inner nuclear layer. The outer retinal layer includes the outer plexiform layer and photoreceptor layer. Measurements of the inner retinal layer (bold double-sided arrows) and outer retinal layer (double-sided arrows) were made 1 mm from the fovea. Among the inner retinal layers, progressive thinning was observed in the nerve fiber layer, and the ganglion cell layer (arrows) and the inner nuclear layer was relatively preserved (arrowheads). E and F show the average retinal thickness values (um) of A and D, respectively. The average retinal thickness values were obtained from the 1, 3, 6 mm thickness map of each quadrant (OCT, Spectralis OCT; Heidelberg Engineering, Heidelberg, Germany). The retinal thickness of the whole macula was markedly decreased one year after the NVG diagnosis

**Fig. 4:**
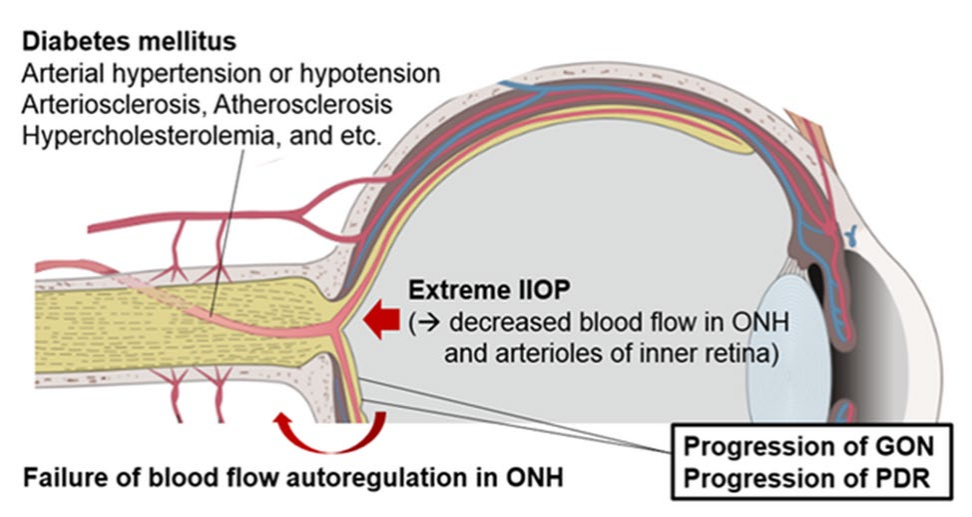
All possible mechanisms related to acute central vision loss after IOP rise due to NVG in patients with PDR. Diabetes mellitus and several other systemic conditions are factors that make NVG patients more susceptible to retinal ischemia. IOP rise due to NVG can accelerate the progression of glaucomatous optic neuropathy and diabetic retinopathy. In addition, especially in group 1, sustained extremely increased IOP may restrict the retinal blood flow through ONH and inner retinal circulation. Since the blood flow autoregulation is effective over only a narrow critical range normally, under the high IOP, the retinal blood flow restriction may not be compensated. The factors mentioned above can affect vision loss in a complex fashion. The Fig. 4 was created by Jong Suk Lee who is one of the co-authors

Park et al. suggested that visual deterioration after NVG in patients with PDR was attributed to the progression of diabetic retinopathy or GON [17]. In addition to these factors, considering the relationship between the period of IOP increase and deterioration of central vision and the representative case (Fig. 3), retinal ischemia due to blood flow restriction during severe IOP rise seems to be a reasonable additional cause of visual deterioration. The probable mechanisms for retinal ischemia due to increased IOP are as follows: (1) decreased blood flow in the optic nerve head (ONH), (2) failure of blood flow autoregulation in the ONH and (3) decreased blood flow in the arterioles and capillaries of the inner retina (Fig. 4). Perfusion pressure decrease and retinal blood flow autoregulation failure due to severe IOP rise inhibit blood flow in ONH [30, 31]. In addition, there have been many reports of retinal artery occlusion due to IOP rise [18, 32]. When perfusion from the optic nerve head is non-physiologically reduced, inner retinal blood circulation may be more directly affected by increased IOP. Moreover, diabetic peripheral neuropathy, the aging process, and accompanying hypertension or arteriosclerosis/atherosclerosis are additional factors that can accelerate retinal ischemia due to increased IOP [33, 34].

Figure 3 shows serial optical coherence tomography (OCT) photographs, which reveal inner retinal edema with hyper-reflectivity and subsequent progressive retinal thinning. These findings are similar to the pattern of incomplete CRAO rather than complete CRAO and support our conclusion that early AGVI can prevent vision loss. It can be explained because the mechanism of retinal ischemia is not due to embolism, but because the blood flow is limited by the decrease in perfusion pressure and blood flow autoregulation failure. Because the mechanism of retinal ischemia is different, unlike in embolic CRAO, where the damage starts at the middle retinal layer, ischemic change was observed mainly in the inner layer adjacent to the eyeball cavity.

Cascades from the increased IOP mentioned above are presumed to be the contributors to vision loss, especially in group 1 (Fig. 4). The reason for delayed AGVI was not only due to patient compliance, including rejection of surgical treatment, personal circumstances, or difficulties undergoing emergency operations but also delayed consulting process from retina specialists who diagnosed NVG to glaucoma specialists who did not have much experience in managing NVG after diabetic vitrectomy. It has been difficult to specify IOP, which causes impaired blood flow in the optic nerve head and retinal circulation. In hyphema patients (otherwise, healthy eye), it is accepted that surgical management should be performed on the second day for 60 mmHg, the fifth day for 50 mmHg, and the seventh day for 35 mmHg [35]. Based on our study results, in NVG patients with advanced PDR, the maximum tolerable period of the retina against ischemia due to medically uncontrolled high IOP of more than 30 mmHg is estimated to be less than one week. The consulting process with glaucoma specialists, medical treatment attempts, and the evaluation of its effectiveness should be completed within one week without delay.

Limitations of this study include its retrospective nature, small sample size, and limited follow-up period. However, it is difficult to conduct a well-designed case-controlled study or prospective randomized trials because NVG after vitrectomy is rare, conducted under different systemic and ocular conditions, and the threat of vision. Our study applied specific exclusion criteria despite having an insufficient number of cases to evaluate visual outcomes and its associated factors. Although long-term outcomes over one year of AGVI were not included in this study’s results, we aimed to investigate the effect of temporary IOP rise on the retina and visual function of PDR patients, so that the appropriate timing of AGVI could be determined. Furthermore, the time point of NVG diagnosis may not be exactly the time point of the IOP increase. However, this study analyzed the effect of the degree and duration of IOP elevation on visual acuity clinically rather than a detailed review of the pathophysiology, ideal treatment options for each stage, and course of NVG. Theoretically, in many cases of group 2, the open-angle served as a potential reservoir for medical treatment. Future research will be required to provide strong evidence for predicting responsiveness to initial non-surgical treatment through the iridocorneal angle evaluation or alternative methods in NVG. In addition, although there was no difference in IOP at the time of NVG, preoperative BCVA showed difference between group 1 and group 2. Media opacity (corneal edema, hyphema, and vitreous hemorrhage), diabetic optic neuropathy or microvascular infarction of macula may be possible factors. A well-designed, large scale study is needed to determine whether these factors can affect the timing and prognosis of AGVI.

In conclusion, AGVI was a safe and effective procedure that enabled successful IOP control in patients with NVG associated with diabetic vitrectomy. However, to improve the visual outcomes of AGVI, physicians should be cautious not to delay surgical intervention, especially in patients who do not respond to initial non-surgical treatment. If IOP is not adequately controlled (< 30 mmHg) within a week from the time of NVG diagnosis, early AGVI within six days might be necessary to preserve useful vision.

## Data Availability

All data generated or analyzed during this study are included in this published article. De-identified data are available from the corresponding author on reasonable request.

## References

[1] Shchomak Z, Cordeiro Sousa D, Leal I, Abegão Pinto L (2019). Surgical treatment of neovascular glaucoma: a systematic review and meta-analysis. Graefes Arch Clin Exp Ophthalmol.

[2] Havens SJ, Gulati V (2016). Neovascular Glaucoma. Dev Ophthalmol.

[3] He Y, Tian Y, Song W, Su T, Jiang H, Xia X (2017). Clinical efficacy analysis of Ahmed glaucoma valve implantation in neovascular glaucoma and influencing factors: a STROBE-compliant article. Medicine.

[4] Hayreh SS (2007). Neovascular glaucoma. Prog Retin Eye Res.

[5] Yap-Veloso MI, Simmons RB, Echelman DA, Gonzales TK, Veira WJ, Simmons RJ (1998). Intraocular pressure control after contact transscleral diode cyclophotocoagulation in eyes with intractable glaucoma. J Glaucoma.

[6] Allen RC, Bellows AR, Hutchinson BT, Murphy SD (1982). Filtration surgery in the treatment of neovascular glaucoma. Ophthalmology.

[7] Hong CH, Arosemena A, Zurakowski D, Ayyala RS (2005). Glaucoma drainage devices: a systematic literature review and current controversies. Surv Ophthalmol.

[8] Yalvac IS, Eksioglu U, Satana B, Duman S (2007). Long-term results of Ahmed glaucoma valve and Molteno implant in neovascular glaucoma. Eye.

[9] Zhang HT, Yang YX, Xu YY, Yang RM, Wang BJ, Hu JX (2014). Intravitreal bevacizumab and Ahmed glaucoma valve implantation in patients with neovascular glaucoma. Int J Ophthalmol.

[10] Zhou M, Xu X, Zhang X, Sun X (2016). Clinical outcomes of Ahmed Glaucoma valve implantation with or without Intravitreal Bevacizumab pretreatment for neovascular Glaucoma: a systematic review and Meta-analysis. J Glaucoma.

[11] Tang M, Fu Y, Wang Y, Zheng Z, Fan Y, Sun X (2016). Efficacy of intravitreal ranibizumab combined with Ahmed glaucoma valve implantation for the treatment of neovascular glaucoma. BMC Ophthalmol.

[12] Liu L, Xu Y, Huang Z, Wang X (2016). Intravitreal ranibizumab injection combined trabeculectomy versus Ahmed valve surgery in the treatment of neovascular glaucoma: assessment of efficacy and complications. BMC Ophthalmol.

[13] Rodrigues GB, Abe RY, Zangalli C, Sodre SL, Donini FA, Costa DC (2016). Neovascular glaucoma: a review. Int J Retina Vitreous.

[14] Sakamoto M, Hashimoto R, Yoshida I, Ubuka M, Maeno T. Risk factors for neovascular glaucoma after vitrectomy in eyes with proliferative diabetic retinopathy. Clinical ophthalmology (Auckland, NZ). 2018;12:2323–9. 10.2147/OPTH.S184959 PMC6241680 30532517

[15] Goto A, Inatani M, Inoue T, Awai-Kasaoka N, Takihara Y, Ito Y (2013). Frequency and risk factors for neovascular glaucoma after vitrectomy in eyes with proliferative diabetic retinopathy. J Glaucoma.

[16] Kwon JW, Jee D, La TY (2017). Neovascular glaucoma after vitrectomy in patients with proliferative diabetic retinopathy. Medicine.

[17] Park UC, Park KH, Kim DM, Yu HG (2011). Ahmed glaucoma valve implantation for neovascular glaucoma after vitrectomy for proliferative diabetic retinopathy. J Glaucoma.

[18] Lee JS, Kim JY, Jung C, Woo SJ. Iatrogenic ophthalmic artery occlusion and retinal artery occlusion.Progress in retinal and eye research. 2020:100848. 10.1016/j.preteyeres.2020.100848 32165219

[19] Liang X, Zhang Y, Li YP, Huang WR, Wang JX, Li X. Frequency and Risk Factors for Neovascular Glaucoma After Vitrectomy in Eyes with Diabetic Retinopathy: An Observational Study. Diabetes therapy: research, treatment and education of diabetes and related disorders. 2019;10(5):1801–9. 10.1007/s13300-019-0644-0 PMC6778559 31321746

[20] Itakura H, Kishi S, Kotajima N, Murakami M (2004). Persistent secretion of vascular endothelial growth factor into the vitreous cavity in proliferative diabetic retinopathy after vitrectomy. Ophthalmology.

[21] Wakabayashi Y, Usui Y, Okunuki Y, Ueda S, Kimura K, Muramatsu D (2012). Intraocular VEGF level as a risk factor for postoperative complications after vitrectomy for proliferative diabetic retinopathy. Investig Ophthalmol Vis Sci.

[22] Chen HJ, Ma ZZ, Li Y, Wang CG (2019). Change of vascular endothelial growth factor levels following vitrectomy in eyes with proliferative Diabetic Retinopathy. J Ophthalmol.

[23] Weinreb RN, Wasserstrom JP, Parker W. Neovascular glaucoma following neodymium-YAG laser posterior capsulotomy. Archives of ophthalmology (Chicago, Ill: 1960). 1986;104(5):730-1. 10.1001/archopht.1986.01050170120035 2423063

[24] Senn P, Schipper I, Perren B (1995). Combined pars plana vitrectomy, phacoemulsification, and intraocular lens implantation in the capsular bag: a comparison to vitrectomy and subsequent cataract surgery as a two-step procedure. Ophthalmic Surg Lasers.

[25] Stefansson E, Landers MB 3rd, Wolbarsht ML. Increased retinal oxygen supply following pan-retinal photocoagulation and vitrectomy and lensectomy. Trans Am Ophthalmol Soc. 1981;79:307–34. PMC1312190 7200671

[26] Chung TY, Chung H, Lee JH (2002). Combined surgery and sequential surgery comprising phacoemulsification, pars plana vitrectomy, and intraocular lens implantation: comparison of clinical outcomes. J Cataract Refract Surg.

[27] Lahey JM, Francis RR, Kearney JJ (2003). Combining phacoemulsification with pars plana vitrectomy in patients with proliferative diabetic retinopathy: a series of 223 cases. Ophthalmology.

[28] Tseng HY, Wu WC, Hsu SY (2007). Comparison of vitrectomy alone and combined vitrectomy, phacoemulsification and intraocular lens implantation for proliferative diabetic retinopathy. Kaohsiung J Med Sci.

[29] Cheng Y, Liu XH, Shen X, Zhong YS (2013). Ahmed valve implantation for neovascular glaucoma after 23-gauge vitrectomy in eyes with proliferative diabetic retinopathy. Int J Ophthalmol.

[30] Hayreh SS (2001). Blood flow in the optic nerve head and factors that may influence it. Prog Retin Eye Res.

[31] Hayreh SS (2009). Ischemic optic neuropathy. Prog Retin Eye Res.

[32] Rooney DM, Oltmanns MH, Sapp MR, Morris RE, NECROSIS OF THE RETINA AND UVEAL TRACT SECONDARY TO EXPANDING GAS TAMPONADE.Retinal cases & brief reports. 2018. 10.1097/ICB.0000000000000841 30601459

[33] Hayreh SS, Bill A, Sperber GO. Effects of high intraocular pressure on the glucose metabolism in the retina and optic nerve in old atherosclerotic monkeys. Graefe’s archive for clinical and experimental ophthalmology = Albrecht von Graefes Archiv fur klinische und experimentelle Ophthalmologie. 1994;232(12):745–52. 10.1007/BF00184278 7890189

[34] Haefliger IO, Meyer P, Flammer J, Lüscher TF (1994). The vascular endothelium as a regulator of the ocular circulation: a new concept in ophthalmology?. Surv Ophthalmol.

[35] Walton W, Von Hagen S, Grigorian R, Zarbin M (2002). Management of traumatic hyphema. Surv Ophthalmol.

